# Predicting the Level of Tumor-Infiltrating Lymphocytes in Patients With Breast Cancer: Usefulness of Mammographic Radiomics Features

**DOI:** 10.3389/fonc.2021.628577

**Published:** 2021-03-11

**Authors:** Hongwei Yu, Xianqi Meng, Huang Chen, Jian Liu, Wenwen Gao, Lei Du, Yue Chen, Yige Wang, Xiuxiu Liu, Bing Liu, Jingfan Fan, Guolin Ma

**Affiliations:** ^1^ Department of Radiology, China-Japan Friendship Hospital, Beijing, China; ^2^ Beijing Engineering Research Center of Mixed Reality and Advanced Display, School of Optics and Photonics, Beijing Institute of Technology, Beijing, China; ^3^ Department of Pathology, China-Japan Friendship Hospital, Beijing, China; ^4^ Department of Ultrasound medicine, China-Japan Friendship Hospital, Beijing, China; ^5^ Peking University China-Japan Friendship School of Clinical Medicine, Beijing, China; ^6^ Graduate School of Peking Union Medical College, Chinese Academy of Medical Sciences and Peking Union Medical College, Beijing, China

**Keywords:** breast cancer, tumor-infiltrating lymphocytes, mammographic, radiomics, machine learning

## Abstract

**Objectives:**

This study aimed to investigate whether radiomics classifiers from mammography can help predict tumor-infiltrating lymphocyte (TIL) levels in breast cancer.

**Methods:**

Data from 121 consecutive patients with pathologically-proven breast cancer who underwent preoperative mammography from February 2018 to May 2019 were retrospectively analyzed. Patients were randomly divided into a training dataset (n = 85) and a validation dataset (n = 36). A total of 612 quantitative radiomics features were extracted from mammograms using the Pyradiomics software. Radiomics feature selection and radiomics classifier were generated through recursive feature elimination and logistic regression analysis model. The relationship between radiomics features and TIL levels in breast cancer patients was explored. The predictive capacity of the radiomics classifiers for the TIL levels was investigated through receiver operating characteristic curves in the training and validation groups. A radiomics score (Rad score) was generated using a logistic regression analysis method to compute the training and validation datasets, and combining the Mann–Whitney U test to evaluate the level of TILs in the low and high groups.

**Results:**

Among the 121 patients, 32 (26.44%) exhibited high TIL levels, and 89 (73.56%) showed low TIL levels. The ER negativity (p = 0.01) and the Ki-67 negative threshold level (p = 0.03) in the low TIL group was higher than that in the high TIL group. Through the radiomics feature selection, six top-class features [Wavelet GLDM low gray-level emphasis (mediolateral oblique, MLO), GLRLM short-run low gray-level emphasis (craniocaudal, CC), LBP2D GLRLM short-run high gray-level emphasis (CC), LBP2D GLDM dependence entropy (MLO), wavelet interquartile range (MLO), and LBP2D median (MLO)] were selected to constitute the radiomics classifiers. The radiomics classifier had an excellent predictive performance for TIL levels both in the training and validation sets [area under the curve (AUC): 0.83, 95% confidence interval (CI), 0.738–0.917, with positive predictive value (PPV) of 0.913; AUC: 0.79, 95% CI, 0.615–0.964, with PPV of 0.889, respectively]. Moreover, the Rad score in the training dataset was higher than that in the validation dataset (p = 0.007 and p = 0.001, respectively).

**Conclusion:**

Radiomics from digital mammograms not only predicts the TIL levels in breast cancer patients, but can also serve as non-invasive biomarkers in precision medicine, allowing for the development of treatment plans.

## Introduction

Breast cancer is the most common malignant tumor among women globally, with a high mortality rate, making early correct diagnosis and effective treatment essential. In recent years, immunotherapy and immune checkpoint blockade (ICB) for the treatment of breast cancer patients have raised concerns in clinical practice (1, 2). However, only a portion of patients respond to current immunotherapy, and predictive biomarkers are necessary for patients who are suitable for immunotherapy (3).

Tumor-infiltrating lymphocytes (TILs) is a promising biomarker; it is now known that the success of ICB-based immunotherapy requires pre-existing anti-tumor immunity (4), which can reflect an individual’s immune tumor response and has strong prognostic and predictive significance (5–7). Increased TIL levels positively correlate with pathological complete response (pCR) and improved patient survival rates, especially in triple-negative breast cancer (TNBC) and human epidermal growth factor receptor 2 (HER 2)-positive breast cancer subtypes. Although the International Immuno-Oncology Biomarker Working Group on Breast Cancer has issued the latest TIL assessment guidelines, the process is still laborious and subjective, with variability between and within raters (8). Therefore, a more objective and reliable method to evaluate TILs in breast tumor is essential.

Radiomics is a recently emerging technique in computational medical imaging and involves extraction and analyses of a large number of quantitative imaging features, such as volume, size, shape, and intensity from medical images. It is different from traditional methods because it converts medical images into mineable high-dimensional data (9, 10). Radiomics can help support patient diagnosis, prognosis, treatment, and prediction in clinical practice (11–13). Magnetic resonance imaging (MRI) plays an important role in the diagnosis of breast cancer, and a few recent studies reported its correlation with the clinical decision among breast cancer patients (14, 15). In addition, several studies have shown that quantitative imaging features from MRI can predict TIL levels and molecular subtypes in patients with breast cancer (16, 17). On the other hand, mammography is a simple, convenient, and low-cost examination without contrast agent injection, compared with MRI. It is widely used in breast cancer screening and diagnosis. Recently, a study demonstrated that quantitative radiomics features derived from mammography can distinguish high and low TILs in patients with TNBC (18). Another study showed that radiomics with mammography can predict breast cancer molecular subtypes (19). However, there are no studies that predict the relationship between TIL levels and breast cancer through mammography. Preoperative evaluation of TILs is a significant biomarker of prognosis and therapeutic response. Therefore, the aim of this study was to evaluate the usefulness of a radiomics model from mammography data in predicting TIL levels in breast cancer patients.

## Materials and Methods

### Patients and Imaging Dataset

This retrospective study was approved by the Institutional Ethics Review Committee of the China–Japan Friendship Hospital, and the requirement for informed consent was waived for all patients. Between February 2018 and May 2019, 121 consecutive patients with breast cancer were enrolled in this study. The inclusion criteria were as follows: (1) unilateral mass type breast cancer was recruited; (2) preoperative bilateral mammography must be performed; (3) having complete clinical data; (4) having complete pathological data, including postoperative immunohistochemical results. All patients received preoperative mammograms through a digital technique using Lorad Selenia (Hologic Gen-Probe, San Diego, USA). The quantization was set to 14-bit for the full-field digital mammographic images with pixel sizes of 70 µm × 70 µm. Images of the craniocaudal (CC) view and the mediolateral oblique (MLO) view were obtained from mammograms of each patient. A total of 121 single masses were analyzed. 121 patients were randomly divided into the training dataset (n = 85) and the validation dataset (n =36) using statistical software.

### Tumor Segmentation and Radiomics Feature Extraction

A radiologist with more than ten years of work experience manually outlined tumor edges in the image. The three-dimensional segmentation of tumor regions of interest (ROIs) was performed using the ITK-SNAP software (version 3.8, Philly, PA, USA), in which the radiological characteristic of the lesion area was extracted. A total of 612 first order shape texture wavelet lbp2d features were extracted using the Pyradiomics software (version 2.2.0, Boston, MA, USA). These radiomics features included texture, morphologic, and statistical features of gray values. Shape, perimeter, area, and size represented the morphological characteristics. Correlation, entropy, contrast, inertia, and homogeneity were the texture features extracted. Finally, the statistical features of gray values involved kurtosis, variance, and gray average.

### Radiomics Feature Selection and Classifier Construction

For the purpose of constructing a predictive model, a one-way analysis of variance (ANOVA) was applied to filter out features with a variance of 0, and then the rest of the radiomics features were retained to select the most relevant features using recursive feature elimination (RFE). According to the Mann–Whitney U test, the top-class features were screened out to build the final logistic regression classifier, which was used to perform radiomics feature selection in the training dataset. Classification performance was evaluated using the area under the receiver operating characteristic curve (AUC). Finally, a radiomics score (Rad score) was developed using the logistic regression model and was used to calculate for the training and validation datasets.

### Evaluation of Tumor-Infiltrating Lymphocytes

After hematoxylin and eosin (H&E) staining, the tumor tissue section was evaluated by a pathologist who had 20 years of professional experience in breast tumor diagnosis. The number of TILs was confirmed by the same pathologist. In order to facilitate the evaluation of variables, we divided the tumor samples into two groups: (1) the low TIL level group was defined as having a TIL density of <50%, and (2) the high TIL level group was defined as having a TIL density >50%. The evaluation criteria followed the latest TIL assessment guidelines issued by the International Immuno-Oncology Biomarker Working Group on Breast Cancer (8).

### Statistical Analysis

All statistical tests were performed using SPSS software (SPSS, version 25, Chicago, IL, USA). A p value **<**0.05 was considered statistically significant. To evaluate the disparity among the patients**’** clinicopathologic characteristics, the chi-square test was used for categorical variables. ANOVA was used to filter out features with a variance of 0. The top-radiological features were correlated with the logistic regression classification using the Mann–Whitney U test. To assess the difference between the predictable competence of the high and low TIL levels based on the training and validation datasets, ROC curves were developed. A Rad score was generated using a logistic regression model to calculate the training dataset and validation dataset.

## Results

### Patients’ Characteristics

The basic characteristics of the patients are shown in **Table 1**. Among the 121 patients, 32 (26.44%) exhibited high TIL levels, whereas 89 (73.56%) showed low TIL levels. The mean ages and menopausal status of patients in the high and low TIL groups were not statistically significant (p = 0.87, p = 0.38), respectively. The ER negativity and the Ki-67 negative threshold level in the low TIL group were higher than that in the high TIL group, and the difference between the two groups was statistically significant (p = 0.01 and p = 0.03, respectively). The patients’ characteristics in the training and validation datasets of this study are listed in **Table 2**. In terms of clinicopathological aspects, there was no statistical significance between the validation and training datasets.

**Table 1:** Clinicopathologic characteristics of patients.

Variables	High TILs level (>50%)	low TILs level(<50%)	*p*-value
	(n = 32)	(n = 89)	
Age, years	50.4 ± 8.9	50.7 ± 11.5	0.87
Menopausal status			0.38
premenopausal	12(37.5%)	26(29.2%)	
postmenopausal	20(62.5%)	63(70.8%)	
Histologic grade			0.81
Grade 1	5(15.6%)	10(11.2%)	
Grade 2	16(50.0%)	47(52.8%)	
Grade 3	11(34.4%)	32(36.0%)	
Pathologic type			0.22
IDC	22(68.8%)	74(83.1%)	
TLC	5(15.6%)	7(7.9%)	
Other	5(15.6%)	8(9.0%)	
ER			0.01
Positive	7(21.9%)	50(56.2%)	
Negative	25(78.1%)	39(43.8%)	
PR			0.67
Positive	21(65.6%)	62(69.7%)	
Negative	11(34.4%)	27(30.3%)	
HER2			0.17
Positive	9(28.1%)	15(16.9%)	
Negative	23(71.9%)	74(83.1%)	
Molecular subtypes			0.67
Luminal A	10(31.3%)	35(39.3%)	
Luminal B	12(37.5%)	26(29.2%)	
HER2-enriched	5(15.6%)	18(20.2%)	
Triple negative	5(15.6%)	10(11.2%)	
Ki-67			0.03
≧14%	5(15.6%)	40(44.9%)	
<14%	27(84.4%)	49(55.1%)	

ER, estrogen receptor; PR, progesterone receptor; HER 2, human epidermal growth factor receptor 2; TILs, tumor-infiltrating lymphocytes.

**Table 2:** Characteristics of patients in the training set and validation sets.

Characteristics	Training set	Validation set	*p-*value
	(n = 85)	(n = 36)	
Age, years	50.6 ± 10.5	52.3 ± 10.5	0.19
Menopausal status			0.79
premenopausal	24(28.2%)	11(30.6%)	
postmenopausal	61(71.8%)	25(69.4%)	
Histologic grade			0.95
Grade 1	20(23.5%)	8(22.2%)	
Grade 2	40(47.1%)	18(50.0%)	
Grade 3	25(29.4%)	10(27.8%)	
Pathologic type			0.42
IDC	72(84.7%)	30(83.3%)	
TLC	10(11.8%)	6(16.7%)	
Other	3(3.5%)	0(0%)	
ER			0.21
Positive	68(80.0%)	25(69.4%)	
Negative	17(20.0%)	11(30.6%)	
PR			0.28
Positive	56(65.9%)	20(55.6%)	
Negative	29(34.1%)	16(44.4%)	
HER2			0.72
Positive	19(22.4%)	7(19.4%)	
Negative	66(77.6%)	29(80.6%)	
Molecular subtypes			0.81
Luminal A	28(32.9%)	11(30.6%)	
Luminal B	37(43.5%)	14(38.9%)	
HER2-enriched	11(12.9%)	5(13.9%)	
Triple negative	9(10.6%)	6(16.7%)	
Ki-67			0.93
≧14%	55(64.7%)	23(63.9%)	
<14%	30(35.3%)	13(36.1%)	

ER, estrogen receptor; PR, progesterone receptor; HER 2, human epidermal growth factor receptor 2.

### Radiomics Feature Selection and Classifier Construction

**Figure 1:**
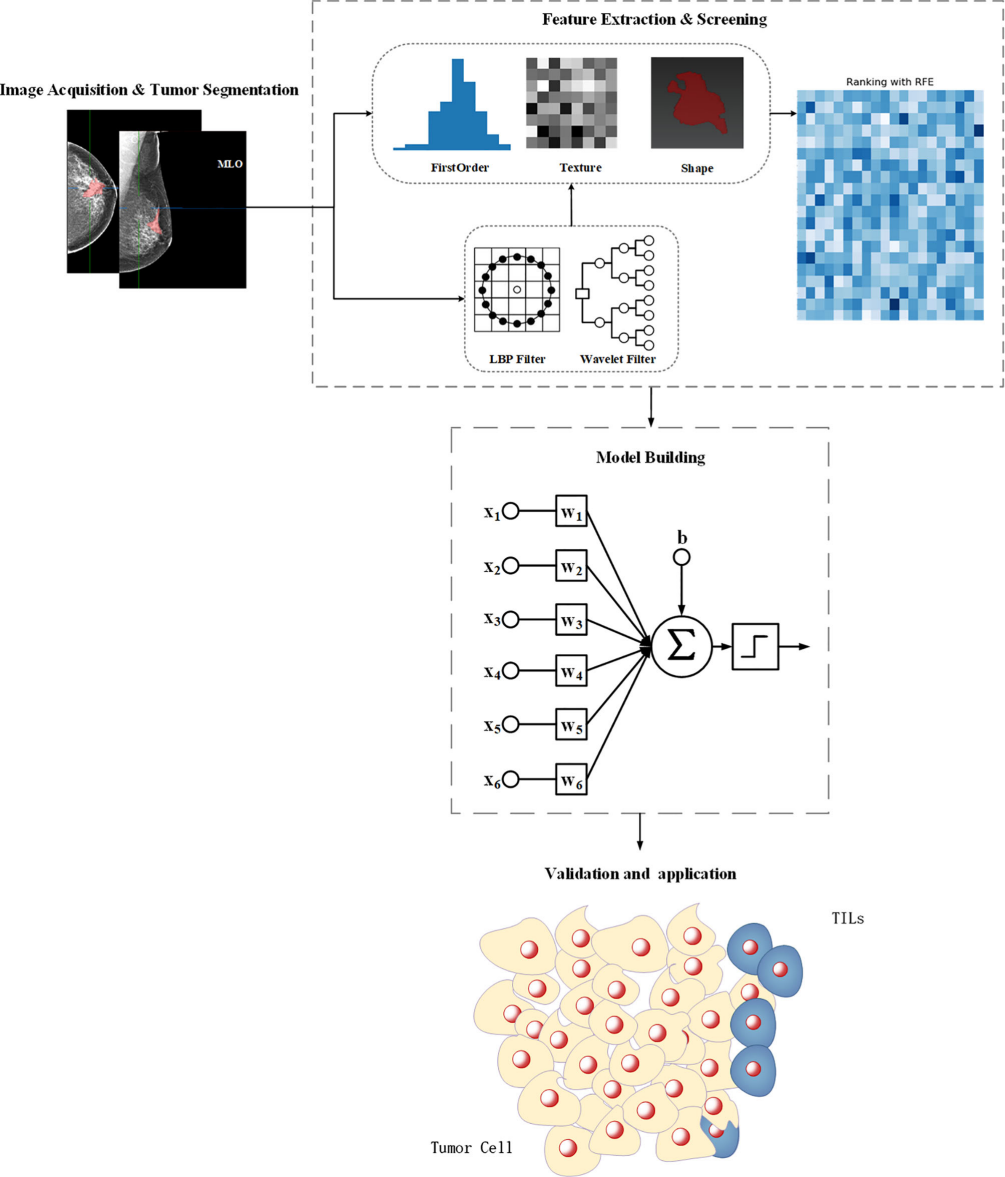
The workflow for feature engineering of mammographic radiomics, which included four main steps. Image acquisition and tumor segmentation; radiomics feature extraction and screening; predictive model building; model validation application.

**Figure 2:**
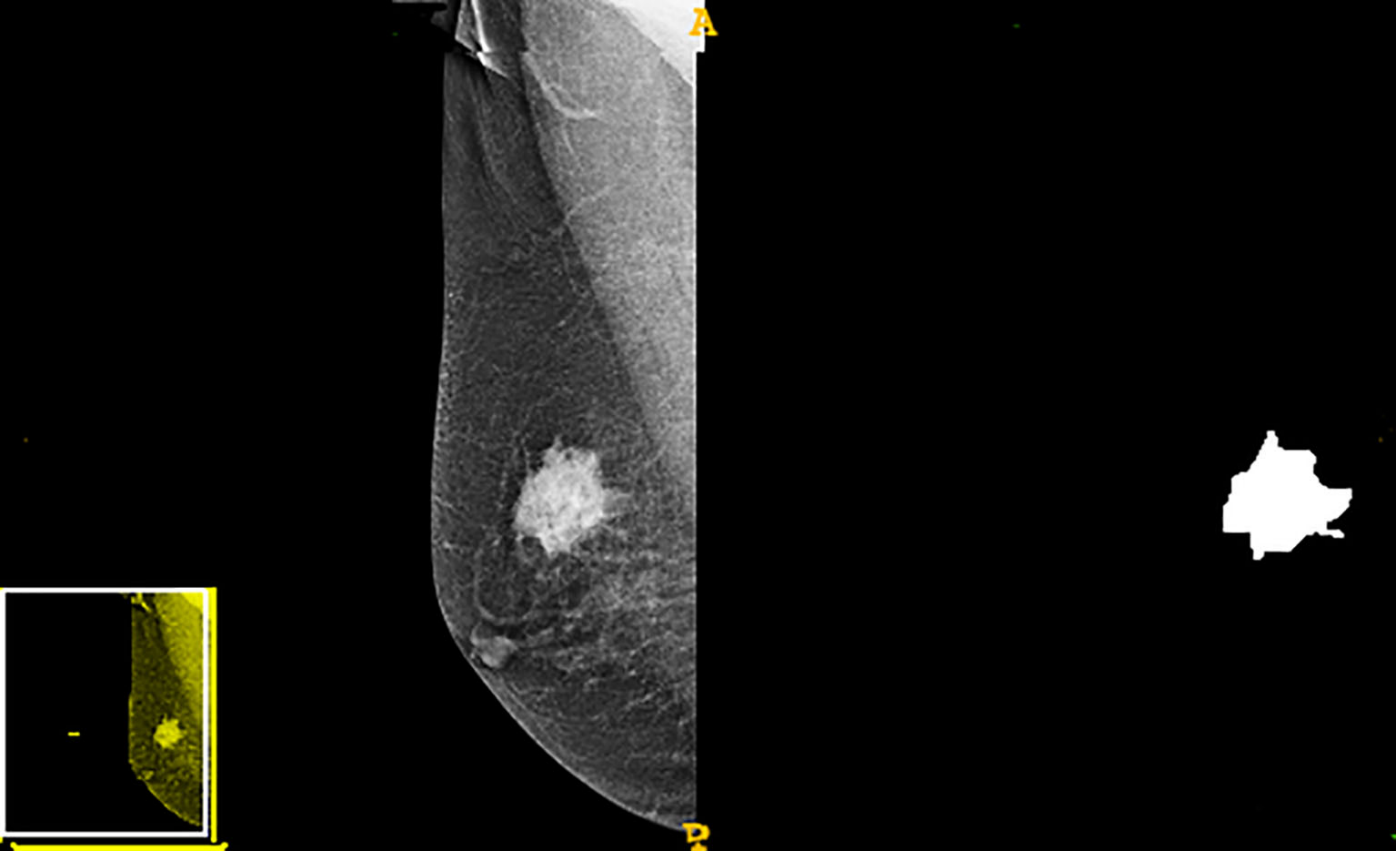
The diagram of the breast tumor segmented.

**Figure 3:**
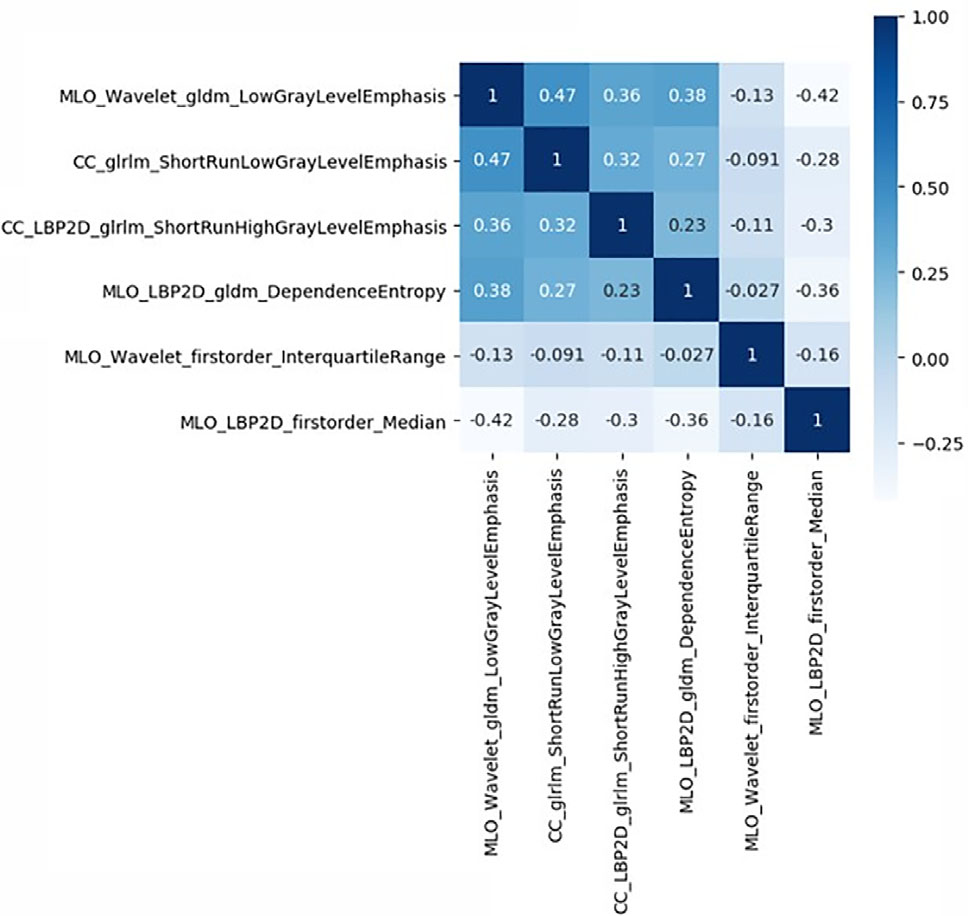
Correlative heatmap between six top-class radiomics features and TIL levels. The values in the square lattices represent the magnitude of R value of correlation analysis displayed by color difference.

The study flowchart is presented in **Figure 1**. A diagram of the breast tumor segment is shown in **Figure 2**. In total, 612 radiomics features that represent quantitative images were extracted from the CC and MLO of mammograms. Through ANOVA, all the features with a variance of 0 were eliminated, and 517 radiomics features remained after the analysis. Subsequently, ten features were selected for further evaluation through RFE, and then the six top-class features with p-values <0.05 were selected using the Mann–Whitney U test in the training set (**Table 3**). **Figure 3** shows that the correlation between the top six features, including wavelet GLDM low gray level emphasis (MLO) (p = 0.018), GLRLM short run low gray level emphasis (CC) (p = 0.005), LBP2D GLRLM short run high gray level emphasis (CC) (p = 0.014), LBP2D GLDM dependence entropy (MLO) (p = 0.008), wavelet interquartile range (MLO) (p = 0.007), and LBP2D median (MLO) (p = 0.017). Finally, the top six features were used to build radiomics classifiers based on logistic regression to predict the TIL level. A Rad score for the training and validation datasets was calculated.

**Table 3:** Analysis of radiomics features between low and high TIL levels in training set.

Radiomics features	Low TILs Level	High TILs Level	*p*-value
	(<50%)	(>50%)	
	(n = 62)	(n = 23)	
Wavelet GLDM low gray level emphasis (MLO)			0.0182
Mean	1.8885e-4	1.2827e-4	
Range	(5.8353e-5–9.2686e-4)	(5.8817e-52–1.1960e-4)	
GLRLM short run low gray level emphasis (CC)			0.0059
Mean	0.0005	0.0003	
Range	(0.0002–0.0014)	(0.00012–0.0005)	
LBP2D GLRLM short run high gray level emphasis (CC)			0.0146
Mean	0.0147	0.0116	
Range	(0.00242–0.0401)	(0.00392–0.0257)	
LBP2D GLDM dependence entropy (MLO)			0.0080
Mean	0.1951	0.1573	
Range	(0.07422–0.4727)	(0.07152–0.3471)	
Wavelet interquartile range (MLO)			0.0070
Mean	1323.3154	1603.0453	
Range	(659.03062–2481.4555)	(742.57562–2754.6258)	
LBP2D median (MLO)			0.0175
Mean	5.1935	5.4348	
Range	(4.02–6.0)	(5.02–6.0)	

CC, craniocaudal; MLO, mediolateral oblique; GLDM, gray level difference matrix; GLRLM, Gray level run length matrix; TILs, tumor-infiltrating lymphocytes.

### Performance and Validation of the Radiomics Classifiers

**Figure 4:**
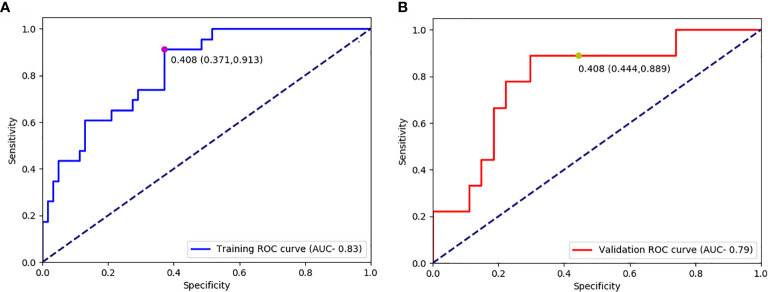
Receiver operating characteristic curves for predicting the TIL levels in the training datasets **(A)** and validation **(B)** datasets.

**Figure 5:**
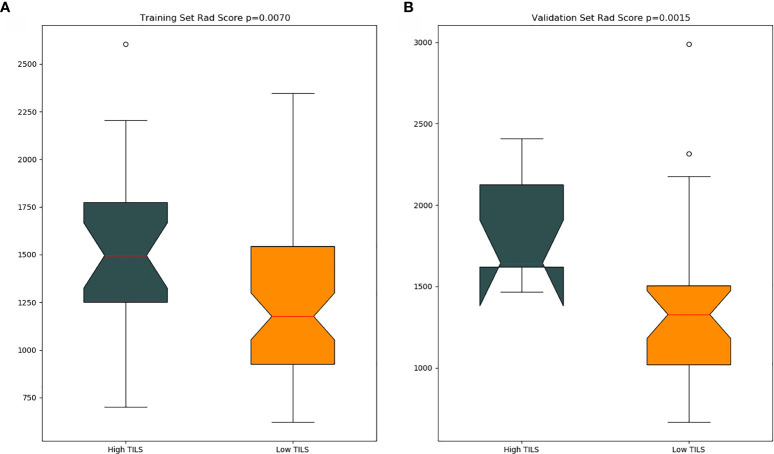
Box plot shows Rad score distributions between the training **(A)** and validation **(B)** groups.

The optimal cut-off value produced through the ROC curve analysis was 0.408. In both the training and validation sets, the radiomics classifier had an excellent performance for classifying the TIL levels. The AUC value was 0.83 (95% confidence interval [CI], 0.738–0.917) in the training dataset and had positive predictive value of 0.913. For the validation set, the classifier had an AUC value of 0.79 (95% CI, 0.615–0.964) and a positive predictive value of 0.889 (**Table 4**, **Figure 4**). The Rad scores for the training and validation sets with respect to the high and low TIL levels are described in **Figure 5**. The predictive performances as determined by Box-plot are presented as statistically different, with p <0.05 (training set p = 0.018, validation set p = 0.031).

**Table 4:** The predictive performance of radiomics classifier in training and validation sets.

Datasets	Cutoff	AUC (95% CI)	SEN	SPE	Accuracy	PPV	NPV
Training	0.408	0.828(0.738, 0.918)	0.913	0.629	0.706	0.477	0.951
Validation	0.408	0.790(0.616, 0.965)	0.889	0.556	0.639	0.400	0.938

AUC, area under curve; 95% CI, 95% confidence interval; SEN, sensitivity; SPE, specificity; PPV, positive predictive value; NPV, negative predictive value.

## Discussion

For TNBC and HER 2-positive breast cancer patients, the TIL levels have a valuable prognostic and predictive ability (20). Many studies have shown that high TIL level is a significant predictor of prognosis and increased pCR rates after chemotherapy (21, 22). Furthermore, the combined application of TIL and clinicopathological criteria can be used to detect and identify early breast cancer patients with better prognosis and avoid unnecessary immunotherapy. Therefore, TILs could serve as useful predictive biomarkers to select patients who could potentially benefit from immunotherapy (1). However, because of the non-uniformity of TIL concentrations in the tumor, the outcome acquired through biopsy may not reflect the whole tumor tissue; the gold standard for evaluating TILs through pathologists’ visual assessment of H&E-stained tumor sections could also be limited mainly by observer diversity. On the other hand, medical imaging plays an irreplaceable role in tumor diagnosis, treatment, and treatment monitoring and is the most useful tool for oncology. Unlike biopsy, imaging is usually widely used in clinical practice because it can be used non-invasively to assess the characteristics of human tissues. Radiomics can extract information-rich imaging functions with high throughput, which is different from traditional subjective imaging and can quantify imaging information that the human eye cannot detect.

Previously, abundant evidence has reported on the relationship between TILs and MRI features. Wu et al. (23) showed that the density of TILs in tumors is closely related to the MRI enhancement form. Fogante et al. (24). used a slightly smaller ROI to assess the relationship between the ADC value and TIL level. Denkert et al. (25) showed that a higher density of TILs is correlated with improved efficacy of neoadjuvant chemotherapy in breast cancer patients, especially when the survival rates of TNBC and HER 2-positive patients are prolonged. However, MRI and mammography have different imaging characteristics, and no research has explored mammographic images in evaluating the status of TILs. Thus, analyzing the characteristics of breast cancer based on the morphology, density, and anatomical features of the mammogram is of great significance for the evaluation of TIL levels. A radiomics method to predict the tumor TIL levels among breast cancer patients was performed in this study. A total of six radiomics features were selected [Wavelet GLDM low gray level emphasis (MLO), GLRLM short-run low gray-level emphasis (CC), LBP2D GLRLM short-run high gray-level emphasis (CC), LBP2D GLDM dependence entropy (MLO), wavelet interquartile range (MLO), and LBP2D median (MLO)]. Wavelet GLDM low gray level emphasis represents the magnitude of a low gray value distribution. The higher the value, the greater the density of the low gray level in the image. GLRLM short run low gray level emphasis measures the joint distribution of shorter run lengths with lower gray level values. The LBP2D GLRLM short run high gray level emphasis measures the joint distribution of shorter run lengths with higher gray level values. LBP2D GLDM dependence entropy means the randomness of GLDM, and a higher dependence entropy implies a more complex texture. The wavelet interquartile range represents P_25_ and P_75_ are the 25^th^ and 75^th^ percentiles of the image array, respectively. The LBP2D median refers to the median gray level intensity within the ROI (26, 27). In mathematics, the GLDM and GLRLM characteristics have different functions and definitions; thus, it has a very good advantage in measuring the heterogeneity of tumor texture features. These texture features based on GLDM and GLRLM are considered adjacent pixels, so it is very suitable for quantization of tumor texture and heterogeneity (28).

Many previous studies have underlined the importance of entropy (29, 30), but one study suspects that entropy is not suitable for the construction of elastic net regression because of the disadvantage of multicollinearity (31). We used the texture features that could interact with each other by combining traditional statistics to build the logical regression classifier. To filter out the coarse feature with redundant, noisy, and irrelevant dimensions, a relatively small subset of the radiomics characteristics was selected. The number of top-class features selected in the prediction model depends on the purpose and the problem to be solved in the process of constructing the classifier (32). In order to improve the accuracy of the predictive model, we combined the Mann–Whitney U test and logical regression classifier to select informative elements. Our results show that a predictive model and the correlation with TIL levels showed excellent discriminative ability among the low and high TIL groups, with AUCs of 0.83 and 0.79 in the training and validation groups, respectively. Despite the limited number of tumor samples in the training and validation sets, the Rad score was able to identify the difference in TIL levels between these datasets. In our study, we observed that the high TIL levels had p-values that were less than that of the low TIL levels, according to the Mann–Whitney U test. The difference was also statistically significant.

Our study has two advantages. First, to the best of our knowledge, this is the first study wherein machine learning has been used to evaluate TIL levels among breast cancer patients. Our study demonstrated that radiomics from qualitative mammographic image characteristics can be used to predict TIL levels. Second, we used standardized texture values to build a logical regression classifier because all texture features had diverse ranges, which could increase the accuracy of predictive modeling.

However, our study also has some limitations. First, this study had a small sample size and was a single-centered retrospective study. Further studies involving multiple centers and a large number of patients are necessary. Second, our radiomics classifier was calculated using ROI drawn only on the single largest slice in the mammographic image, which may increase concerns regarding selection bias. Third, we did not perform an external validation to confirm the effectiveness of our findings, which may lead to differences. In the future, a larger subset of the dataset is needed for validation. Finally, we could not contrast the manifestation of mammograms and DC-EMR images in this study. However, mammography remains the most common method for breast cancer screening and diagnosis. This study aimed to investigate the predictive capacity between radiological features of mammograms and TIL levels in breast cancer patients. If radiomics predictive modeling from mammograms has excellent performance in evaluating TIL levels, more valuable information will be provided to radiologists and clinicians, which can help radiologists and clinicians to make better clinical decisions for breast cancer patients.

In conclusion, a radiomics predictive model from digital mammogram images was found to be a useful method for discriminating low and high TIL levels in patients with breast cancer. Such a quantitative radiomics predictive model is an efficient, non-invasive, and cost-effective method to predict TIL levels in patients with breast cancer and could facilitate the development of non-invasive biomarkers in precision medicine, as well as the development of a treatment plan.

## Data Availability Statement

The raw data supporting the conclusions of this article will be made available by the authors, without undue reservation.

## Ethics Statement

The studies involving human participants were reviewed and approved by the Institutional Ethics Review Committee of the China–Japan Friendship Hospital. The patients/participants provided their written informed consent to participate in this study.

## Author Contributions

The acquisition, analysis, data explanation, and manuscript draft were finished by HY. XM is responsible for the analysis and explanation of the radiomics imaging features data. HC analyzed and explained the pathological analysis. JL, WG, LD, YC, YW, XL, and BL acquired the clinical information and revised the manuscript. JF and GM designed the study and made multiple revisions to the manuscript. All authors contributed to the article and approved the submitted version.

## Conflict of Interest

The authors declare that the research was conducted in the absence of any commercial or financial relationships that could be construed as a potential conflict of interest.
